# Results of Response-Guided Therapy with Pegylated Interferon Alpha 2a in Chronic Hepatitis B and D

**DOI:** 10.3390/tropicalmed9040073

**Published:** 2024-03-30

**Authors:** George S. Gherlan, Stefan D. Lazar, Augustina Culinescu, Dana Smadu, Andreea R. Vatafu, Corneliu P. Popescu, Simin A. Florescu, Emanoil Ceausu, Petre I. Calistru

**Affiliations:** 1 Infectious Diseases Department, Universitatea de Medicina si Farmacie ”Carol Davila”, 050474 Bucuresti, Romaniapcalistru@yahoo.com (P.I.C.); 2 Infectious Diseases Department, Spitalul Clinic de Boli Infectioase si Tropicale ”Dr. Victor Babes”, 030303 Bucuresti, Romania

**Keywords:** hepatitis D, pegylated interferon alpha, response guided, virologic response, HDV RNA, treatment

## Abstract

Pegylated interferon alpha 2a continues to be used for the treatment of chronic hepatitis D. The reported on-treatment virologic response varies between 17 and 47%, with relapses in more than 50% of these patients. No stopping rules have been defined, and the duration of the treatment is not clearly established, but it should be between 48 and 96 weeks. In total, 76 patients with compensated liver disease treated with peg-interferon according to the Romanian National protocol for the treatment of hepatitis D were retrospectively included. The duration of treatment was up to 96 weeks, with the following stopping rules: less than a 2 log HDV RNA decrease by week 24 and less than a 1 log decrease every 6 months afterwards. Six months after stopping the treatment, it can be restarted for unlimited cycles. The inclusion criteria were aged above 18, HBs Ag-positive, HDV RNA detectable, ALT above ULN and/or liver fibrosis at least F1 at liver biopsy, or Fibrotest and/or Fibroscan higher than 7 KPa and/or inflammation at least A1 at liver biopsy or Fibrotest. We monitored our patients for a total period of 4 years (including those that repeated the cycle). After the first 6 months of treatment, 27 patients (35.5%) had a greater than 2 log HDV RNA decrease, 19 of them achieving undetectable HDV RNA. Seventeen patients (22.3%) had undetectable HDV RNA 24 weeks after stopping 96 weeks of treatment, and none relapsed in the following 2 years. Of these 17 patients, 6 were cirrhotic, and 4 had F3. Undetectable HDV RNA at 24 weeks was the only parameter that predicted a long-term suppression of HDV RNA. In 49 patients, the treatment was stopped after 6 months according to protocol, but it was restarted 6 months later. Five of these patients finished a 48-week course of treatment; none achieved undetectable HDV RNA. During the first course of therapy, 45 patients had at least one moderate adverse reaction to treatment. In one patient, the treatment was stopped due to a serious adverse event (osteomyelitis). Treatment doses had to be reduced in 29 patients. The virologic response at week 24 can select the patients who will benefit from continuing the treatment from those who should be changed to another type of medication when available.

## 1. Introduction

Hepatitis D virus (HDV) is a defective RNA virus that requires the presence of the hepatitis B virus (HBV) to fulfil its lifecycle. Hepatitis D infection can be established as a coinfection in a patient not previously infected with HBV or superinfection in a patient with a chronic HBV infection. The host usually resolves coinfection by eliminating both agents, while superinfection most frequently results in chronic HBV and HDV infection [1]. Acute HDV infection in either one of its previous forms can cause acute liver failure, while chronic infection is the most aggressive form of viral infection currently affecting humans, with frequent and rapid evolution to liver cirrhosis and its complications [2]. Current treatment options are limited. Until recently, pegylated interferon alpha (PEG-INF-alfa) was the only available drug that showed a degree of efficacy in the treatment of this form of hepatitis, but this degree was low, and the side effects were not negligible [3]. Interferons are proteins naturally produced by the human immune system when stimulated by diverse aggressions, especially by viruses and bacteria but also parasites or tumour cells [4]. Interferons have proven antiviral activity against many viruses, including HBV and hepatitis C virus (HCV); they have direct antiviral activity but also play an immunomodulatory role [5]. Successful treatment with PEG-INF-alfa occurs in patients in which both HBV and HDV virologic markers drop, and this suggests that a combined action on both infections is required to obtain a favourable result [2,6]. The concrete activity of PEG-INF-alfa on HDV is not completely understood. Several mechanisms have emerged from many in vitro studies. They are proposed as possible mechanisms of action: inhibition of replication in chronically infected hepatocytes, a possible effect on viral entry, and suppression of cell division-mediated HDV spread by possibly eliminating HDV intermediate replicates during mitosis [2]. The results of the studies show large variations, especially when looking at the viral response. This is probably due to a lack of standardised HDV RNA tests [7,8]. Studies conducted between 2006 and 2019 [8,9,10,11,12,13,14,15,16,17] showed rates of responses between 17% and 47% at the end of treatment with lengths between 48 and 96 weeks. The Hep-Net-International Delta Hepatitis Intervention Trial (HIDIT) I study showed no use of adefovir addition to PEG-INF-alfa 2a and a sustained viral response at week 24 of 28% of the patients treated [16]. The addition of tenofovir was also not useful for significantly increasing the final results in HIDIT II. Still, this study showed a 23% rate of HDV RNA non-detectability at the end of the 6-month follow-up period after 96 weeks of treatment with PEG-INF-alfa 2a alone or with tenofovir [17]. It has been shown in further studies that up to 50% of the patients who end their treatment with undetectable HDV RNA may develop virologic failure in the next ten years [18,19]. The prevalent genotype for HDV in Romania is genotype 1. New drugs for treating HDV infection are under development or already approved in some regions: bulevirtide, lonafarnib, and lambda interferon are in the most advanced phases of development and will be discussed in this article. The main aim of this study was to evaluate whether a response-guided treatment with PEG-INF-alfa could identify patients who would benefit from the pegylated interferon treatment and those for whom this treatment is not beneficial. A secondary aim was to identify the predictive factors for long-term virologic suppression.

## 2. Materials and Methods

In total, 76 patients treated according to the National Romanian protocol for treating HBV/HDV coinfection who started their treatments during 2018 and 2019 were included, and their data were retrospectively collected. The protocol for the treatment of HBV/HDV coinfected patients at that time had the following inclusion criteria:

1. Age over 18 years;
2. Chronic HBV infection documented by HBs Ag positivity for more than 24 weeks before starting the treatment;
3. HDV coinfection proved by HDV Ab positivity and detectable HDV RNA;
4. ALT above the upper limit of normal.

The following exclusion criteria were also used:

1. HCV present co-infection (HCV RNA detectable at start of treatment. Patients treated and with SVR were accepted.);
2. HIV co-infection;
3. Any known cause of immune suppression;
4. Any contraindications for the use of PEG-Interferon alpha 2a, including:
  - Hypersensitivity to PEG-Interferon alpha 2a,
  - Autoimmune diseases,
  - Hepatic decompensation (Child-Pugh B or C class cirrhosis), currently or in the past,
  - Any ongoing known malignancies (solid or haematological),
  - Low platelet number (below 90.000/mm^3^),
  - Baseline neutrophil count below 1500/mm^3^,
  - Any concomitant treatment that would contra-indicate or lower the efficacy of PEG-Interferon alpha 2a;
5. Chronic kidney disease with estimated glomerular filtration rate below 60 mL/min/1.73 m^2^;
6. Pregnancy or pregnancy planning in the following 120 weeks after the start of the treatment;
7. Not being able to sign informed consent or to follow the treatment as prescribed;
8. Any uncontrolled psychiatric disorder;
9. Use of any HDV (experimental) treatment in the previous 3 months before the start of the treatment;
10. Previous PEG-Interferon alpha 2a treatment;
11. Any other contraindication as considered by the investigator.

The exclusion criteria were partly necessary due to the use of interferon treatment (criteria 3, 4, 5, 6, 7, 8). They were verified for all patients included in the treatment according to National Romanian protocol or were verified when collecting the data (criteria 1, 2, 9, 10, 11) and were meant to eliminate the bias from our analysis as much as possible. 

The treatment, according to the protocol, was response-guided with the following stopping rules imposed:

1. Stop the treatment if the HDV RNA at 24 weeks of treatment did not drop with 2 logs compared to the baseline HDV RNA;
2. Stop the treatment if the HDV RNA at 48 weeks of treatment did not drop with 1 log compared to 24 weeks of HDV RNA;
3. Any increase in the HDV RNA compared to the previously determined value led to stopping the treatment.

The treatment consisted of PEG-Interferon alpha 2a (tradename Pegasys®, from Roche, Basel, Switzerland), and the standard dose was 180 mcg administered subcutaneously once weekly. The dose was subject to change according to the insert of the product. The patients were monitored at least monthly (more frequently if necessary, according to the clinical judgement of the physician) in order to identify any significant adverse events that could require dose modification or discontinuation of the treatment. Every 24 weeks, the HDV RNA was determined to verify the above stopping rules.

**Figure 1:**
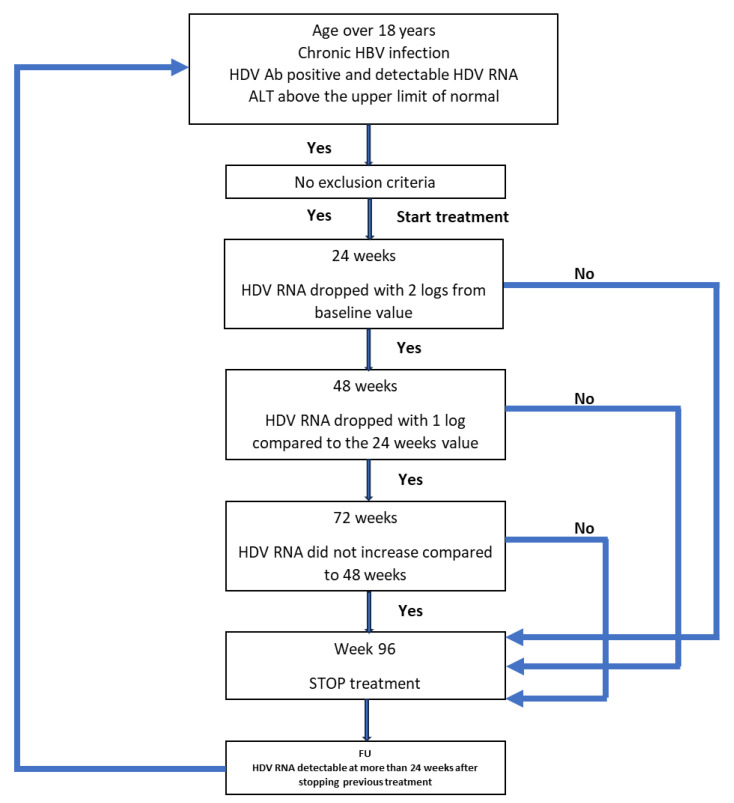
Schematic representation of the protocol used for the treatment of the patients.

If the treatment had to be stopped at any time and for any reason, the patient continued to be monitored. If HDV RNA was detectable 24 weeks after stopping the treatment, the patient could restart the treatment if they fulfilled the inclusion criteria. All patients’ data were monitored for a period of 4 years (192 weeks) from the start of the first treatment, regardless of the outcome. The protocol is schematically represented in Figure 1. 

HDV RNA and HBV DNA were determined in our laboratory using Bosphore® HDV quantitation detection kits, respectively, Cobas®/Roche HBV Tests. 

## 3. Results

### 3.1. Baseline Characteristics 

The study included 45 men and 31 women. Table 1 synthesises their baseline characteristics. 

Thirteen patients had HBV DNA values higher than 20,000 IU/mL, while in the other 63, HBV was suppressed. Only four patients had positive HBeAg, and in all these, the HBV DNA was high. According to the noninvasive methods used (Fibroscan and Fibrotest), 16 patients had cirrhosis, 18 were F3 corresponding to Metavir stage, 7 were F2, and 35 were F0/F1. 

At baseline, 10 patients had normal ALT values (below 55 IU/L, according to our laboratory). 

### 3.2. On Treatment Evolution

After the first 6 months of treatment, 27 patients (35.5%) had a higher than 2 log decrease in HDV RNA compared to baseline. Only these patients were eligible to continue their treatment. The rest of the 49 patients stopped their dose, and we continued monitoring the disease. At this time point, 19 of the 27 patients had already reached HDV RNA non-detectability. 

Of those who had a normal ALT at baseline, only six maintained normal values at 6 months. The other four that had an increase in ALT had a non-satisfactory virologic response, in three of them with an increase from baseline and in one with a non-significant decrease. Another 14 patients obtained normal ALT values at 6 months. Of the total of 20 patients with normal values at 6 months, only one had to stop the treatment according to stopping rules, because they did not obtain a significant decrease in the HDV RNA (more than 2 logs) from baseline. 

At week 48, three more patients achieved HDV RNA non-detectability. However, only 24 out of the 27 patients from the previous timepoint could continue the treatment. as in three patients, the HDV RNA did not show a further decrease greater or equal to 1 log. At this time point (week 48), 18 patients had normal ALT values, and they were among those continuing the treatment. After the other 24 weeks, at week 72 of treatment, 21 patients (27.6%) had undetectable HDV RNA, and only these were eligible to continue the treatment for another 24 weeks, until week 96, as in the other three, the viral load increased. At week 72, the same 18 patients as at week 48 had normal ALT values and were among those continuing the treatment. We did not check the HDV RNA at week 96 because, according to the protocol, patients could restart the treatment only after a 6-month pause.

**Figure 2:**
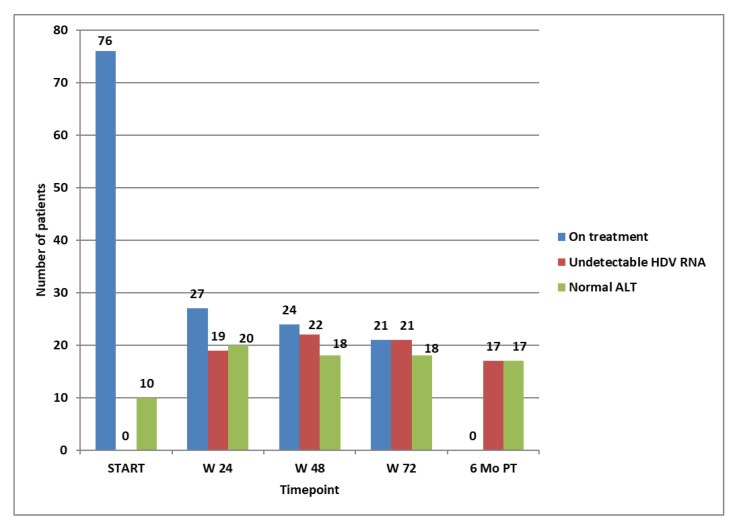
Patients on treatment at selected time points, patients with not-detectable HDV RNA, and patients with normal ALT at selected time points.

Six months after the treatment, of the 21 patients that achieved non-detectability of HDV RNA at week 72, only 17 (22.3%) remained non-detectable, all having normal ALT values. None of these relapsed at the end of the monitoring period (96 weeks after stopping a successful course of 96 weeks of treatment). All 17 patients who remained non-viremic after 96 weeks from stopping the treatment obtained non-detectability of HDV RNA after the first 24 weeks of treatment. Of these long-term responders, six had cirrhosis, and four had an F3 corresponding to the Metavir stage of fibrosis at baseline. The evolution of the entire group for the first course of treatment is shown in Figure 2. 

**Figure 3:**
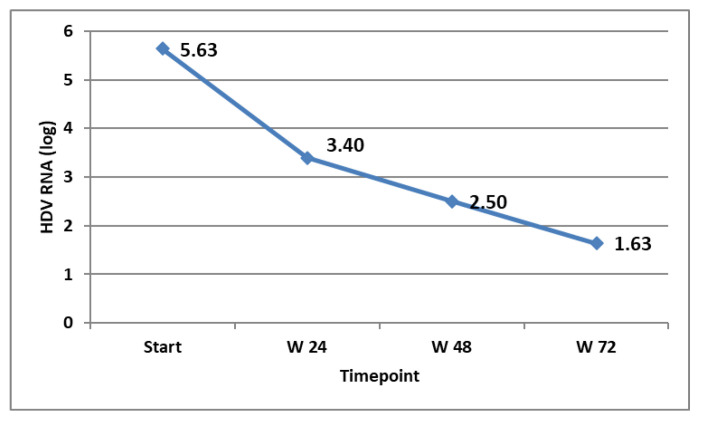
HDV RNA (expressed in log) at different timepoints in the patients on treatment.

**Figure 4:**
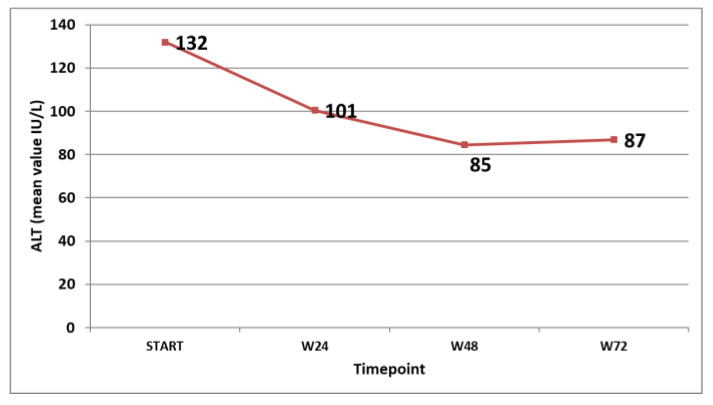
Mean ALT values (IU/L) at different timepoints in the patients on treatment.

The virological response in the patients that were on treatment at the specific timepoints was followed and is shown in Figure 3. The biochemical response (ALT decline and normalisation) was also monitored and is shown in Figure 4.

Almost all patients experienced at least one grade 1 (according to Common Terminology Criteria for Adverse Events (CTCAE) version 5.0) adverse event (68 out of 76, 89.4%). Forty-five patients experienced at least one grade 2 CTCAE v5.0 event (59.2%). Ten patients experienced grade 3 or 4 CTCAE adverse events. Nineteen patients required dose reduction to the first level (135 mcg/week), and 10 patients required reduction to the second level (90 mcg/week) or had to skip one dose. Only one patient required permanent dosing discontinuation because of a severe adverse event (SAE grade 3 or higher CTCAE v5.0), and this event was osteomyelitis. All patients who continued their treatment for more than 48 weeks experienced at least one adverse event, most frequently fatigue, headache, or dizziness. The exact frequency of the adverse events and their types are shown in Table 2.

According to the abovementioned stopping rules, 49 patients had to stop the treatment after the first 24 weeks of treatment because of the lack of significant virologic response (defined as a decline with 2 logs compared to baseline). Out of these 49, 43 patients started a second round of treatment. Only five of these met the criteria of significant virologic response. They followed a course of a total of 48 weeks of treatment when they had to stop because of the complete lack of virologic response between weeks 24 and 48. 

No HBsAg or HBeAg loss was noted during the monitoring period in this study. 

## 4. Discussions

Although progress has been made in the field of chronic HBV/HDV coinfection treatment, pegylated interferon alpha 2a remains an option [2,4]. Its association with other medications could be the key to controlling the infection or even resolving it in our patient, at least until other more potent and efficient drugs are available.

There are two main types of responses we target during our patients’ treatment: a virological response (defined variably from study to study, either as a significant decrease in HDV RNA at the selected reference timepoint or, in some cases, as complete non-detectability at the same timepoint) and a biochemical response (also defined variably from study to study, either as a significant decrease in liver transaminases or, more frequently, as their normalisation). 

Studies have shown different success rates, with quite a large variability, between them (from 17% to 47% virological response at the end of the follow–up period). This large variability is caused by many factors: the variable period of treatment between studies (48 to 96 weeks), the variable period of follow–up (6 to 36 months), the addition of other drugs (nucleoside or nucleotide analogues active on HBV), the use of non-validated HDV RNA kits, and the variable definition of virological response. An HIDIT II study showed that the occurrence of HDV RNA is possible even after two years of non-detectability [17]; so, this should not be considered healing but, until further evidence, long-term suppression. 

According to a recent meta-analysis [20], the biochemical response is reached in about 33%, with variations between 27% and 40%. 

The addition of other available medicines (bulevirtide or lonafarnib) is promising, as it increases both types of responses and also the combined outcome. Bulevirtide is a newly approved drug, the first in its class, an entry-inhibitor for HBs receptor. Bulevirtide can be used alone or in combination with pegylated interferon. HBsAg loss occurred only in patients treated with the combination therapy. Real-life studies confirm the results of clinical trials, with faster virological efficacy with the combination than with BLV monotherapy. Lonafarnib is a prenylation inhibitor acting on farnesyl transferase, already used for the treatment of a rare form of progeria. The results were also best in the groups of patients treated with lonafarnib in combination with pegylated interferon alpha 2a. [21] Nucleic acid polymers and pegylated lambda interferon are other possible future options [21] 

No response-guided study on the treatment of HDV/HBV infection with pegylated interferon alpha 2a has been published to date. Our study has the advantage of a complete follow-up of the patients for four years and two years of monitoring after the end of the treatment. Also, the number of patients included in the study is significant. 

As shown by our results, obtaining an early virological complete response (HDV RNA not detectable at 24 weeks) is the most important predictor for long-term suppression after two years of treatment. 

Our study showed a complete virological response in 21 patients (27.6%) at week 72, but 4 of these patients (19%) relapsed 6 months after stopping the treatment. A total of 17 patients had a long-term suppression of viral load (22.3%). ALT normalisation occurred in 18 out of the 24 patients at week 72 (75%), and all 17 patients who obtained long-term suppression of viremia maintained normal levels of ALT until the end of the monitoring period.

Most of the patients described adverse events during the treatment period. Dizziness, fever, fatigue, and headache were the most frequent. Some of the adverse events, such as headache, fatigue, insomnia, irritability, neutropenia, and low platelet count, became more frequent with time, while some occurred mostly at the beginning of treatment (fever, site injection reactions, myalgias, and bone/joint pain). Twenty-nine patients required treatment dose reduction or temporary interruption of dosing during the treatment, while only one had to stop the treatment during a serious adverse event.

## 5. Conclusions

The present study shows that using a response-guided protocol selects the patients with the best chances to obtain a long-term suppression of HDV RNA based on the HDV RNA decline after the first 24 weeks of treatment. Considering that a new alternative treatment is available (bulevirtide), which is efficient in obtaining a biochemical response with much fewer adverse effects, this response-guided indication could be part of a two-step treatment protocol for choosing candidates for long treatment with PEG-INF alfa2a versus bulevirtide alone or in combination.

## Figures and Tables

**Table 1:** Baseline characteristics of the patients. Age—expressed in years. BMI—Body Mass Index—expressed in kg/m^2^. ALT—Alanine aminotransferase—expressed in IU/L. AST—Aspartate aminotransferase. Platelet count—expressed in platelets/mm^3^. AFP—alfafetoprotein—expressed in ng/ml. INR—International Normalised Ratio. Fibrotest, actitest—numeric results of the respective tests measuring fibrosis and inflammation. Fibroscan—expressed in kilopascals.

Parameter	Minimum	Maximum	Mean	STDEV
**AGE**	20	76	44	14.9
**BMI**	16.8	36.8	26.2	4.2
**ALT**	30	576	132	101
**AST**	25	194	68	37
**Platelet count**	90,000	347,000	184,530	54,982
**AFP**	1.4	70	8.77	14.75
**INR**	0.88	2.08	1.08	0.23
**Fibrotest**	0.1	1	0.488	0.292
**Actitest**	0.25	0.94	0.88	0.51
**Fibroscan**	5.8	27	11.3	5.24
**HDV RNA (log)**	3	8.23	5.63	1.58
**HBV DNA (log)**	1.3	8.31	2.73	1.61

**Table 2:** Adverse events occurred during specific intervals (expressed as a percentage of the total number of patients on treatment during that specific interval).

Side Effect	Baseline–24 Weeks (N = 76) (%)	24–48 Weeks N = 27 (%)	48–72 Weeks N = 24 (%)	72–96 Weeks N = 21 (%)
**Systemic Manifestations or Injection Site Events**				
Fatigue	50	51.8	66.6	61.9
Headache	47.3	62.9	58.33	76.1
Dizziness	81.6	74	75	76.1
Fever	63.1	48.1	45.8	33.3
Nausea	42.1	70.3	62.5	33.3
Loss of appetite	13.1	18.5	12.5	9.5
Site injection reaction	22.3	11.1	12.5	9.5
**Psychiatric Disorders**				
Insomnia	28.9	40.7	45.8	42.85
Depression	22.3	29.6	16.66	14.2
Irritability	30.2	40.7	50	52.3
**Musculoskeletal Symptoms**				
Myalgia	43.4	37	29.1	23.8
Bone/joint pain	22.3	22.2	20.8	14.2
**Skin or Hair Disorders**				
Hair loss	22.3	29.6	33.3	38
Pruritus	14.5	11.1	8.33	4.7
Rash	5.2	7.4	4.1	4.7
**Hematologic or Biochemical Reactions**				
Neutropenia	22.3	29.6	29.1	28.5
Low platelet count	19.7	25.9	25	28.5
ALT increase	6.5	11.1	12.5	14.2

## Data Availability

Data are available from the corresponding author.
